# Associations of blood glucose, helper T cells and cytokine levels with degree of periodontal lesion in type 2 diabetes mellitus patients accompanied by chronic periodontitis

**DOI:** 10.4314/ahs.v23i2.27

**Published:** 2023-06

**Authors:** Cheng Wang, Bing Zhou

**Affiliations:** Department of Stomatology, The 900th Hospital of Joint Logistic Support Force, People's Liberation Army, Fuzhou 350025, Fujian Province, China

**Keywords:** Type 2 diabetes mellitus, chronic periodontitis, blood glucose, helper T cell, cytokine, periodontal lesion

## Abstract

**Background:**

To explore the associations of blood glucose with degree of periodontal lesions in patients with type 2 diabetes mellitus (T2DM) accompanied by chronic periodontitis (CP).

**Methods:**

Sixty-five eligible patients were included as a T2DM+CP group, another 65 patients with T2DM alone were included as a T2DM group, and another 65 patients with CP alone were included as a CP group. Their blood glucose, insulin, Th cells and cytokine levels and periodontal indices were compared. The correlations between each index and periodontal indices were analysed. The influencing factors for T2DM accompanied by CP were explored.

**Results:**

The levels of fasting plasma glucose (FPG), glycated hemoglobin A1c (HbA1c), fasting insulin (FINS) and homeostasis model assessment-insulin resistance (HOMA-IR) of T2DM+CP, T2DM and CP groups followed a descending order (P<0.05). FPG, HbA1c, FINS, CD4+ Th1 cell, CD4^+^ Th17 cell, interferon-gamma (IFN-γ) and interleukin-17 (IL-17) all had positive correlations with gingival index, bleeding index, probing depth and attachment loss in T2DM patients accompanied by CP (P<0.05). Periodontal lesions were more severe in T2DM patients accompanied by CP, and the severity was positively correlated with the levels of FPG, HbA1c, Th1, Th17, IFN-γ and IL-17.

**Conclusion:**

High levels of FPG, HbA1c, IFN-γ and IL-17 are independent risk factors for T2DM accompanied by CP.

## Introduction

As a common chronic metabolic disorder, type 2 diabetes mellitus (T2DM) is mainly manifested as elevation in fasting plasma glucose (FPG) and glycated hemoglobin A1c (HbA1c) levels, accompanied by insulin resistance and hyposecretion. Long-term hyperglycemia easily induces micro-inflammatory response and various complications including oral diseases. Besides, abnormal glucose metabolism greatly increases the risk of periodontitis. In T2DM patients, the prevalence rate of periodontitis is far higher than that of normal people, and the rate and extent of damage to periodontal supporting tissues significantly increase^1^. DM is a risk factor directly leading to the increased morbidity rate of periodontitis and tooth loss rate^2^. After the periodontal tissues of patients with periodontitis are destroyed, harmful bacteria in the periodontal pocket enter the bloodstream, which can cause immuno-inflammatory response, result in islet B-cell injury or destruction, and aggravate DM. About 60% of patients simultaneously suffer from DM and periodontitis^3^. DM and periodontitis have a bidirectional relationship, for which immuno-inflammatory response is the common pathological basis. Periodontal pathogens can mediate immune and inflammatory responses through altering the function of cluster of differentiation 4^+^ (CD4^+^) helper T (Th) cells, and thus play key roles in the onset and progression of periodontitis^4^. However, the associations of blood glucose, Th cell and cytokine levels with the degree of periodontal lesions in T2DM patients accompanied by chronic periodontitis (CP) have seldom been reported hitherto. In this study, therefore, the levels of blood glucose, Th cells and cytokines in T2DM patients accompanied by CP were analysed, and their associations with the degree of periodontal lesion were explored, aiming to provide valuable evidence for clinical diagnosis and treatment.

## Materials and Methods

### Diagnostic criteria

The diagnostic criteria for T2DM were as follows: According to the Guidelines for the Prevention and Treatment of Type 2 Diabetes Mellitus in China^5^, there are symptoms of diabetes mellitus, and the 2-hour glucose tolerance test or random blood glucose ≥11.1 mmol/L (200 mg/dL), or FPG ≥7.0 mmol/L (126 mg/dL), or the measured values still reach the above levels after repeated measurement twice for the cases without typical symptoms.

The diagnostic criteria for CP were as follows: According to the fourth edition of *Periodontics^6^*, there are dental plaques and calculi, the probing depth (PD) of at least two teeth in different quadrants is no less than 5 mm, the attachment loss (AL) is no less than 2 mm, and obvious alveolar resorption is shown by imaging.

### Subjects

This study has been approved by the ethic committee of our hospital, and written informed consent has been obtained from all patients. A total of 65 T2DM patients accompanied by CP diagnosed and treated in our hospital from May 2016 to July 2020 were included as a T2DM+CP group, another 65 patients with T2DM alone were included as a T2DM group, and another 65 patients with CP alone were included as a CP group.

**The inclusion criteria were as follows: 1**) Patients meeting the diagnostic criteria for T2DM and CP, 2) those with at least 10 natural teeth left in the mouth, except for the third molar, 3) those who were injected with insulin or orally given hypoglycemic drugs recently, 4) those without the history of periodontal or orthodontic treatment in the past 6 months, 5) those without using antibiotics, non-steroidal anti-inflammatory drugs, glucocorticoids or immunomodulatory drugs in the past 3 months, 6) those without lesions of oral mucosa or salivary gland, and 7) those with complete clinical data.

**The exclusion criteria were as follows: 1**) Patients with total dentition defect, 2) those with severe malocclusion, 3) those with severe infectious diseases, cardio-cerebrovascular diseases, blood system diseases, dysfunction of heart, liver, lung or kidney, or trauma, 4) those with the history of smoking or drinking, 5) those who could not receive periodontal examination due to other reasons, 6) those with mental disease, or 7) pregnant or lactating women.

### Measurement of blood glucose and insulin levels

After the patients were deprived of food and water for 12 h, 3 mL of fasting venous blood was drawn in the morning and placed in test tubes without anticoagulant. Then FPG level was detected by the glucose oxidase method using 7600 biochemical analyzers (Hitachi, Japan). HbA1c level was detected with high-pressure liquid chromatography using UARIANT II glycosylated hemoglobin analyzer (Bio-Rad, USA). Fasting insulin (FINS) level was measured using AXSYM chemiluminescence system (Abbott, USA). Homeostasis model assessment-insulin resistance (HOMA-IR) was calculated by: HOMA-IR = FPG (mmol/L) ×FINS (mU/L)/22.5.

### Detection of peripheral blood Th1, Th2 and Th17 cells

After the patients were deprived of food and water for 12 h, 2 mL of fasting venous blood was drawn in the morning, placed in heparin-anticoagulant tubes, lysed with red blood cell lysis buffer for 5 min and centrifuged. After the supernatant was discarded, cell suspension was prepared using 1 mL of flow cytometry staining buffer. Then 100 uL of cell suspension was taken, and added FITC-CD4 antibody and Triton respectively for reaction in dark for 30 min. Interferon-gamma (IFN-γ)-PE, interleukin-4 (IL-4)-PE and IL-17-PE monoclonal antibodies were added respectively for reaction in dark for 20 min, with IgG-PE antibody as the isotype control. Finally, the percentages of CD4^+^ Th1 cells, CD4^+^ Th2 cells and CD4^+^ Th17 cells were calculated using a flow cytometer.

### Detection of Th-related cytokines in the peripheral blood and gingival crevicular fluid

Th-related cytokines in peripheral blood and gingival crevicular fluid samples were detected. The gingival crevicular fluid was collected as follows. After the supragingival plaque and dental calculi were removed, aseptic cotton was used to keep the moisture, and the surface was wiped dry using sterile cotton balls. The dental face was blown dry using a pneumatic gun. Then #30 absorbent paper was gently inserted into the periodontal pocket on the side of cheek until there was slight resistance. After 30 s, the paper was taken out and placed into sterile tubes. The same operation was performed at an interval of 30 s. If there was visible blood or saliva contamination, the paper was discarded, and the sample was re-taken after 30 s. Gingival crevicular fluid samples were cryopreserved. Then the levels of IL-2, IL-4, IL-10, IL-17, tumor necrosis factor-β (TNF-β) and IFN-γ were determined using human IL-2 ELISA kit (ab174444), human IL-4 ELISA kit (ab215089), human IL-10 ELISA kit (ab46034), human IL-17 ELISA kit (ab119535), human TNF-β ELISA kit (ab229202) and human IFN-γ ELISA kit (ab46025) (Abcam, USA).

### Examination of periodontal indices

Gingival index (GI): Each index tooth was detected at 6 sites (mesial, median and distal gingival papillae) on the buccal and lingual sides. 0 point: Normal gingiva. 1 point: Mild gingival inflammation, only mild redness and swelling, and no effusion. 2 points: Obvious gingival inflammation, redness and swelling or small ulcers, and a little effusion. 3 points: Severe gingival inflammation, dark red colour, pyorrhea and secretions. The values of the 6 sites were averaged.

Bleeding index (BI): A blunt probe was gently inserted into the gingival sulcus or periodontal pocket, and then taken out. After 30 s, gingival bleeding was observed. 0 point: Healthy gingivae. 1 point: no bleeding on probing, but with changes in gingival colour. 2 points: Punctate gingival bleeding on probing. 3 points: Bleeding on probing and spreading along gingival margins. 4 points: Bleeding on probing, overflowing from the gingival sulcus. 5 points: Spontaneous gingival bleeding.

PD: It refers to the distance from the gingival margin to the bottom of the gingival sulcus or periodontal pocket. Each index tooth was detecte at 6 sites (mesial, median and distal gingival papillae) on the buccal and lingual sides, and the values were averaged. AL: If there is no gingival recession, AL = distance between the enamel-cementum junction and gingival margin - PD. If there is gingival recession, AL = distance between the enamel-cementum junction and gingival margin + PD.

Periodontal examination was conducted by two professional dentists under natural light, and the data were recorded and checked by two intern dentists. The same batch of examination instrument was used, and consistent criteria were employed (*Kappa* >0.8).

### Statistical analysis

SPSS16.0 software (IBM Inc., USA) was used for statistical analysis. Quantitative data were expressed as mean ± standard deviation (-x ± s). First, one-way analysis of variance was used for analysis among groups. In the case of statistically significant differences, the post hoc LSD-t test was performed for comparison between two groups. Count data were expressed as n (%), and the χ^2^ test was performed for comparison. The correlations of blood glucose, insulin, Th cells and related cytokines with periodontal indices in T2DM patients accompanied by CP were explored by Pearson's analysis. Multivariate logistic regression analysis was conducted for the influencing factors for T2DM accompanied by CP. The test level was set as α=0.05, and P<0.05 suggested statistically significant difference.

## Results

### Blood glucose and insulin levels

There were no significant differences in gender and age among different groups (P>0.05). The levels of FPG, HbA1c, FINS and HOMA-IR of T2DM+CP, T2DM and CP groups followed a descending order, and there were significant differences between any two groups (P<0.05) (Table 1).

**Table 1:** Blood glucose and insulin levels

Group	n	Gender (n, %)		Age (year, x̅ ± s)	FPG (mmol/L, x̅ ± s)	HbA1c (%, x̅ ± s)	FINS (mmol, x̅ ± s)	HOMA-IR (x̅ ± s)
							
		Male	Female					
T2DM+CP	65	36	29	46.85±9.37	10.53±1.12	9.00±0.92	11.94±1.23	5.85±1.07
T2DM	65	34	31	47.03±9.42	9.78±1.08^a^	7.95±1.21^a^	11.36±1.03^a^	5.04±0.94^a^
CP	65	35	30	46.92±9.38	4.74±0.32^a^^b^	5.12±0.97^a^^b^	8.24±0.81^a^^b^	1.81±0.29^a^^b^
F		0.124		0.086	16.637	9.583	6.528	24.472
*P*		0.940		0.917	0.000	0.000	0.000	0.000

a P<0.05 *vs*. T2DM+CP group

b P<0.05 *vs*. T2DM group

### Proportions of Th1, Th2 and Th17 cells in the peripheral blood

No significant difference was found in the proportion of Th2 cells among different groups (P>0.05). The proportions of Th1 and Th17 cells had no significant difference between T2DM and CP groups (P>0.05). The proportions of Th1 and Th17 cells in the T2DM+CP group were higher than those of other two groups (P<0.05) (Table 2).

**Table 2:** Proportions of Th1, Th2 and Th17 cells in the peripheral blood

Group	n	Th1 (%, x̅ ± s)	Th2 (%, x̅ ± s)	Th17 (%, x̅ ± s)
T2DM+CP	65	4.52±0.37	0.28±0.10	1.87±0.28
T2DM	65	3.45±0.25^a^	0.31±0.14	0.81±0.15^a^
CP	65	3.37±0.26^a^	0.29±0.12	0.77±0.15^a^
F		10.274	1.306	12.453
*P*		0.000	0.172	0.000

a P<0.05 *vs*. T2DM+CP group.

### Th-related cytokines in the peripheral blood and gingival crevicular fluid

There were no significant differences in the levels of IL-2, TNF-B, IL-4 and IL-10 in the peripheral blood and gingival crevicular fluid among different groups (P>0.05). The levels of IFN-γ and IL-17 in the peripheral blood and gingival crevicular fluid had no significant differences between T2DM and CP groups (P>0.05). The levels of IFN-γ and IL-17 in the peripheral blood and gingival crevicular fluid of the T2DM+CP group were higher than those of other two groups (P<0.05) (Table 3).

**Table 3:** Th-related cytokines in the peripheral blood and gingival crevicular fluid

Group	n	IL-2 (pg/mL, x̅ ± s)		IFN-γ (pg/mL, x̅ ± s)		TNF-β (pg/mL, x̅ ± s)	
		
		Peripheral blood	Gingival crevicular fluid	Peripheral blood	Gingival crevicular fluid	Peripheral blood	Gingival crevicular fluid
T2DM+CP	65	7.03±0.81	1.05±0.34	3.57±0.58	1.83±0.76	1.94±0.82	0.86±0.35
T2DM	65	6.98±0.79	0.97±0.32	1.47±0.41^a^	1.17±0.54^a^	1.88±0.79	0.82±0.33
CP	65	6.95±0.78	1.01±0.36	1.45±0.39^a^	1.14±0.55^a^	1.89±0.81	0.80±0.32
F		0.564	0.873	14.314	5.293	0.351	1.017
*P*		0.537	0.602	0.000	0.000	0.726	0.308

Group	n	IL-4 (pg/mL, x̅ ± s)		IL-10 (pg/mL, x̅ ± s)		IL-17 (pg/mL, x̅ ± s)	
		
		Peripheral blood	Gingival crevicular fluid	Peripheral blood	Gingival crevicular fluid	Peripheral blood	Gingival crevicular fluid
T2DM+CP	65	3.78±1.04	1.63±0.59	1.82±0.75	0.91±0.59	8.93±0.93	3.07±0.78
T2DM	65	3.75±1.02	1.58±0.56	1.77±0.73	0.88±0.57	5.55±0.73^a^	1.52±0.49^a^
CP	65	3.69±1.01	1.60±0.57	1.79±0.72	0.84±0.55	5.54±0.68^a^	1.48±0.46^a^
F		0.524	0.484	0.376	0.731	14.526	13.417
*P*		0.613	0.602	0.815	0.498	0.000	0.000

a P<0.05 *vs*. T2DM+CP group.

### Periodontal indices

GI, BI, PD and AL were higher in the T2DM+CP group than those in the CP group, and they were also higher in T2DM+CP and CP groups than those in the T2DM group, showing significant differences between any two groups (P<0.05) (Table 4).

**Table 4:** Periodontal indices

Group	n	GI (point, x̅ ± s)	BI (point, x̅ ± s)	PD (mm, x̅ ± s)	AL (mm, x̅ ± s)
T2DM+CP	65	1.92±0.26	3.85±0.39	5.25±0.44	4.45±0.25
T2DM	65	0.43±0.07^a^	1.84±0.20^a^	1.98±0.26^a^	0.13±0.08^a^
CP	65	1.01±0.14^a^^b^	2.73±0.28^a^^b^	3.20±0.32^a^^b^	2.38±0.17^a^^b^
F		28.105	22.634	33.168	78.549
*P*		0.000	0.000	0.000	0.000

a P<0.05 *vs*. T2DM+CP group

b P<0.05 *vs*. T2DM group

### Correlations of blood glucose, insulin, Th cells and Th-related cytokines with periodontal indices in T2DM patients accompanied by CP

FPG, HbA1c, FINS, Th1, Th17, IFN-γ and IL-17 all had positive correlations with periodontal indices GI, BI, PD and AL in T2DM patients accompanied by CP (P<0.05) (Table 5).

**Table 5:** Correlations of blood glucose, insulin, Th cells and Th-related cytokines with periodontal indices in T2DM patients accompanied by CP

Index	FPG	HbA1c	FINS	Th1	Th17	IFN-γ	IL-17
GI *r*	0.609	0.709	0.620	0.443	0.575	0.683	0.584
*P*	<0.0001	<0.0001	<0.0001	0.0002	<0.0001	<0.0001	<0.0001
BI *r*	0.502	0.622	0.483	0.486	0.550	0.688	0.583
*P*	<0.0001	<0.0001	<0.0001	<0.0001	<0.0001	<0.0001	<0.0001
PD *r*	0.428	0.643	0.294	0.265	0.539	0.488	0.613
*P*	0.0004	<0.0001	0.0176	0.0328	<0.0001	<0.0001	<0.0001
AL *r*	0.383	0.245	0.608	0.384	0.353	0.389	0.432
*P*	0.0016	0.0488	<0.0001	0.0016	0.0040	0.0013	0.0003

### Influencing factors for T2DM accompanied by CP

Among the variables with statistically significant differences, HOMA-IR was no longer involved in multivariate logistic regression analysis due to its interaction effect with FPG and FINS. Then HbA1c, Th1, Th17, IFN-γ and IL-17 were included into multivariate analysis as the independent variables and assigned as follows: with the median as the cut-off value, FPG: >10.40 mmol/L=1, <10.40 mmol/L=0; HbA1c: >9.10%=1, <9.10%=0; FINS: >12.10 mmol/L=1, <12.10 mmol/L=0; Th1: >4.59%=1, <4.59%=0; Th17: >1.96%=1, <1.96%=0; IFN-γ: >3.90 pg/mL=1, <3.90 pg/mL=0; IL-17: >8.85 pg/mL=1, <8.85 pg/mL=0. Moreover, the diseases in T2DM patients accompanied by CP were used as the dependent variables and assigned as follows: T2DM accompanied by CP=1, T2DM or CP alone=0. The results of logistic regression analysis revealed that high levels of FPG, HbA1c, IFN-/span>γ and IL-17 were independent risk factors for T2DM accompanied by CP (P<0.05) (Table 6).

**Table 6:** Influencing factors for T2DM accompanied by CP

Factor	β	SE	Wald	*P*	OR	95%CI
FPG	1.475	1.256	2.837	0.012	2.063	1.548~3.297
HbA1c	1.824	1.637	2.419	0.009	3.275	2.036~4.852
FINS	1.536	1.198	1.765	0.327	1.984	0.552~2.369
Th1	2.149	1.764	3.281	0.083	2.436	0.384~3.725
Th17	0.965	0.739	1.632	0.145	1.772	0.497~2.863
IFN-γ	2.483	2.167	4.075	0.004	4.365	2.458~5.792
IL-17	2.602	2.354	3.683	0.007	4.718	3.029~5.841

## Discussion

There is a mutual effect between DM and periodontal disease, and they are high risk factors for each other. Periodontitis has been listed as the sixth main complication of DM^7^. Long-term high-glucose state in DM patients easily induces the production of oxygen free radicals, and causes direct damage to islet B-cells and periodontal tissues. After the oxidative stress signalling pathway is activated, the produced inflammatory cytokines activate osteoclast nuclear collagenase, further destroying bone and periodontal tissues. A variety of inflammatory factors released during the onset of periodontitis can damage or destroy islet B-cells, and reduce the activity of insulin receptors in tissues or cells, thereby resulting in insulin resistance and raising the risk of DM. Likewise, this study showed that the levels of FPG, HbA1c, FINS and HOMA-IR had significant differences among T2DM+CP, T2DM and CP groups.

Th cells can be divided into three subgroups (Th1, Th2 and Th17), which belong to CD4+ T lymphocytes. In the case of microbial infectious diseases, Th cell population regulates the immune response, and also plays a supporting role in the differentiation of other immune cells. Th1 cell population resists intracellular pathogen infection mainly through secreting cytokines IL-2, TNF-B and IFN-γ, thereby mediating the inflammatory response. Th2 cell population is involved in humoral immune response mainly through secreting IL-4 and IL-10. Through secreting highly inflammatory cytokine IL-17, Th17 promotes the inflammatory response and mediates autoimmune diseases. Th1- and Th17-related cytokines are involved in the aggravation of periodontal tissue inflammation and alveolar bone destruction, while Th2-related cytokines protect periodontal tissues and mitigate lesions^8^. Deng *et al.* reported that the proportions of Th1 and Th17 cells in the T2DM+CP group were obviously higher than those in CP and control groups, while the proportion of Th2 cells was similar to that in the CP group but obviously lower than that in the control group^9^. Meanwhile, the level of Th17-related cytokine IL-17 significantly rose in the plasma and gingival crevicular fluid, while the level of Th1-related cytokine IFN-γ increased only in the plasma. The levels of other cytokines hardly changed. Thus, the change trend of cytokines is basically the same as that of Th cells. In this study, no control group was set, but a T2DM group was added. The results showed that the level of IFN-γ markedly increased in both plasma and gingival crevicular fluid, which may be attributed to the differences between grouping of s/span>subjects, detection reagents and determination criteria. However, the findings of this study and previous literatures all demonstrate that in T2DM patients accompanied by CP, the Th2-dominated protective immune response is transformed into Th1-dominated destructive immune response, thereby aggravating the Th1/Th2 balance disorder and systemic inflammation. Besides, Th17 cell population may lead to the activation of neutrophil-related cytokines and osteoclasts through secreting and releasing IL-17, thus further worsening the alveolar bone destruction and resorption.

Periodontal indices GI, BI, PD and AL can be used to reflect the severity of periodontal inflammation. The incidence rate and severity of periodontitis in DM patients are far higher than those in non-DM patients^10^, being consistent with the periodontal indices measured in this study. Costa *et al.* reported that the severity of CP was closely related to the increase of HbA1c level^11^. Additionally, Chen et al. found that IFN-γ was widely distributed in the lymphocyte infiltration area of lamina propria of gingival connective tissue, which was positively correlated with the degree of periodontitis^4^. Zhou *et al.* found that the expression of IFN-γ in gingival tissues was remarkably higher in the periodontitis group than those in gingivitis and healthy groups, which rose with increasing PD^12^. Moreover, Thorbert Mros et al. found that the mRNA expression level of IL-17 in the periodontitis group was significantly higher than that in the gingivitis group^13^. Lester et al. reported that the level of IL-17 was positively correlated with the number of gingival sites of AL in patients with CP^14^. In this study, the correlations of FPG, HbA1c, FINS, Th1, Th17, IFN-γ and IL-17 with peri-odontal indices GI, BI, PD and AL in T2DM patients accompanied by CP were consistent with the above studies. Finally, logistic regression analysis revealed that high levels of FPG, HbA1c, IFN-γ and IL-17 were independent risk factors for T2DM accompanied by CP (P<0.05), which was in agreement with a previous study^15^.

In conclusion, periodontal lesions are more severe in T2DM patients accompanied by CP, and the severity is positively correlated with the levels of FPG, HbA1c, Th1, Th17, IFN-γ and IL-17. High levels of FPG, HbA1c, IFN-γ and IL-17 are independent risk factors for T2DM accompanied by CP. Therefore, it is necessary to consider the mutual influence between T2DM and CP, and to simultaneously control the blood glucose level and inflammatory response, aiming to effectively improve the treatment outcomes.
